# Patient-reported outcomes in a trial of exenatide and insulin glargine for the treatment of type 2 diabetes

**DOI:** 10.1186/1477-7525-4-80

**Published:** 2006-10-11

**Authors:** Kristina Secnik Boye, Louis S Matza, Alan Oglesby, Karen Malley, Sunny Kim, Risa P Hayes, Robert Brodows

**Affiliations:** 1 Eli Lilly and Company, Indianapolis, IN 46285, USA; 2 Center for Health Outcomes Research at UBC, Bethesda, MD 20814, USA; 3 Malley Research Programming, Inc., Rockville, MD, USA; 4 School of Public Health, Florida International University, USA

## Abstract

**Background:**

Patient-reported measures can be used to examine whether drug differences other than clinical efficacy have an impact on outcomes that may be important to patients. Although exenatide and insulin glargine appear to have similar efficacy for treatment of type 2 diabetes, there are several differences between the two treatments that could influence outcomes from the patient's perspective. The purpose of the current study was to examine whether the two drugs were comparable as assessed by patient-reported outcomes using data from a clinical trial in which these injectable medications were added to pre-existing oral treatment regimens.

**Methods:**

Patients were randomized to either twice daily exenatide or once daily insulin glargine during a 26-week international trial. At baseline and endpoint, five patient-reported outcome measures were administered: the Vitality Scale of the SF-36, The Diabetes Symptom Checklist – Revised (DSC-R), the EuroQol EQ-5D, the Treatment Flexibility Scale (TFS), and the Diabetes Treatment Satisfaction Questionnaire (DTSQ). Change from baseline to endpoint was analyzed within each treatment group. Group differences were examined with General linear models (GLMs), controlling for country and baseline scores.

**Results:**

A total of 549 patients with type 2 diabetes were enrolled in the trial, and current analyses were conducted with data from the 455 per protocol patients (228 exenatide and 227 insulin glargine). The sample was primarily Caucasian (79.6%), with slightly more men (55.2%) than women, and with a mean age of 58.5 years. Paired t-tests found that both treatment groups demonstrated statistically significant baseline to endpoint change on several of the health outcomes instruments including the DSC-R, DTSQ, and the SF-36 Vitality subscale. GLMs found no statistically significant differences between groups in change on the health outcomes instruments.

**Conclusion:**

This analysis found that both exenatide and insulin glargine were associated with significant improvements in patient-reported outcomes when added to oral medications among patients with type 2 diabetes. Despite an additional daily injection and a higher rate of gastrointestinal adverse events, treatment satisfaction in the exenatide group was comparable to that of the glargine group, possibly because of weight reduction observed in patients treated with exenatide.

## Background

In clinical trials, patient-reported outcome measures can complement clinical outcomes by providing information beyond traditional efficacy and safety measures. When new treatments have comparable efficacy, patient-reported instruments can be used to examine whether drug differences other than clinical efficacy have an impact on outcomes that may be important to patients [1]. Two injectable treatments for patients with type 2 diabetes, insulin glargine and exenatide, have been found to have comparable efficacy as measured by HbA_1c _reduction in a recent 26-week randomized controlled trial [2]. When added to oral medications in this trial, both exenatide and insulin glargine reduced HbA_1c _levels by 1.1%. Insulin, in conventional and analog forms, is a commonly used treatment for such patients [3,4]. Insulin glargine is a long-acting analog with absorption kinetics that provides a relatively consistent basal insulin supplied for approximately 24 hours [5,6]. Exenatide is a recently approved medication that elicits several of the glucoregulatory actions of glucagon-like peptide-1, an incretin hormone that is an essential regulator of normal glucose homeostasis [2,7-14]. Exenatide has post-parandial and fasting blood glucose effects [2]. Although exenatide and insulin glargine appear to have similar efficacy for reduction of HbA_1c_, there are several differences between the two treatments that could influence outcomes from the patient's perspective. Therefore, the purpose of the current study was to conduct a secondary analysis of clinical trial data to examine whether the two drugs were comparable as assessed by patient-reported outcomes.

One difference between these two medications that could lead to differences in patient-reported outcomes is that they have different effects on patients' body weight. Whereas insulin is associated with increased risk of weight gain [15-17], exenatide has repeatedly been found to be associated with weight reduction [7,8,11,12,14]. For example, in a 26-week head-to-head clinical trial, insulin glargine-treated patients had a mean body weight increase of 1.8 kg from a baseline mean of 88.3 kg, whereas exenatide-treated patients decreased in body weight by 2.3 kg from a baseline mean of 87.5 kg [2]. Weight reduction is likely to lead to positive health outcomes for many patients as it has been shown to improve glycemic control and reduce long-term health risks [16,18-21]. Furthermore, lower weight has been found to be associated with greater patient-reported treatment satisfaction and health-related quality of life (HRQL) among patients with diabetes [22-24]. HRQL can be defined as the patient's subjective perception of the impact of health status on physical, psychological, and social functioning [1,25].

Exenatide and insulin also differ in side effect profiles. In clinical trials, the most frequent adverse events reported by patients with exenatide have been gastrointestinal side effects, such as nausea and to a lesser extent vomiting, that tend to occur early in treatment [2,7,8,11,12,14]. These gastrointestinal symptoms are generally found to be mild-to-moderate, and they have only a negligible contribution to the weight effects of exenatide [26,27]. Patients treated with insulin glargine have reported a lower incidence of these side effects [2]. Another difference between the two drugs involves dose frequency. Insulin glargine is administered once per day, whereas exenatide is administered twice per day. In general, reduced dose frequency is thought to be associated with greater treatment satisfaction, although there are exceptions for some patients, diseases, and medications [28-32]. To assess the potential impact of these differences between exenatide and insulin glargine, the current study analyzed change in five patient-reported outcome measures, using data from a clinical trial in which the two drugs had comparable efficacy [2]. These outcome measures assessed HRQL, treatment satisfaction, vitality, treatment flexibility, and impact of diabetes symptoms.

## Methods

### Data source

Data from a 26-week, multicenter, comparator-controlled, open-label, randomized, two-arm, clinical trial were used for this analysis. Data were collected in 13 countries (Australia, Belgium, Brazil, Finland, Germany, Norway, Poland, Portugal, Puerto Rico, Spain, Sweden, the Netherlands, and the United States). All patients were required to have type 2 diabetes that was inadequately controlled with orally administered sulfonylurea and metformin (i.e., HbA_1c _between 7.0% and 10.0%). Patients were randomized to add one of two injectable medications to their oral treatment regimen: exenatide (taken twice-daily, 15 minutes before morning and evening meals; fixed dose of 5 micrograms bid for the first 4 weeks and subsequently increased to 10 micrograms bid) or insulin glargine (forced titration to FBS target ≤ 5.5 mmol/L; administered once daily at bedtime). The oral medications were maintained at pre-study dose levels unless patients experienced hypoglycemia, in which case a 50% reduction in sulfonylurea dose was recommended. The primary objective of the study was to test the hypothesis that glycemic control, as measured by change in HbA_1c_, achieved with exenatide is non-inferior to that of insulin glargine. The current secondary analysis was conducted to compare the two treatment groups with respect to change in patient-reported health outcomes measures. Clinical findings, dropout rates for each treatment group, reasons for study withdrawal, and further description of the trial design are published elsewhere [2].

### Measures

In this trial, patients completed five health outcomes instruments at baseline (week 0) and endpoint (week 26), including two generic and three condition-specific measures. Because generic and condition-specific measures have different strengths, it is often recommended to include both types of instruments in clinical trials [33-36]. Compared with generic measures, the primary advantage of condition-specific measures is that they are frequently found to be more responsive to treatment-related change [37]. An advantage of generic PROs is that they can be used to compare among various populations, make comparisons to the general population, and estimate the relative impact of various medical conditions or treatments [1,38-40]. In addition, generic measures usually assess impact of disease and treatment on overall functioning or a broader range of health domains than condition-specific measures [34,39].

### Diabetes Symptom Checklist – revised (DSC-R)

The DSC-R is a revised version of the DSC-2, which was developed to measure both the frequency and perceived discomfort of physical and psychological symptoms associated with type 2 diabetes and its potential complications [41]. On the 34 items of the DSC-R, participants first indicate whether they have experienced each symptom in the past month by circling "yes" or "no". If "yes" is selected, the participant proceeds to rate the perceived discomfort of the symptom on a 5-point scale ranging from 1 (not at all) to 5 (extremely). When participants report not having the symptom, the item is scored as zero. The instrument yields a total score and the following subscales: Fatigue, Cognitive, Pain, Sensory, Cardiology, Ophthalmology, Hypoglycemia, and Hyperglycemia. Higher scores indicate greater symptom burden. The total score and all dimension scores range from 0 to 5, with higher scores indicating greater discomfort. The DSC-2 has been found to have good internal consistency reliability, test-retest reliability, construct validity, and responsiveness [41,42]. No published literature on the psychometric properties of the DSC-R was located.

### Diabetes Treatment Flexibility Scale (TFS)

The TFS is comprised of 10 items from the 142-item Diabetes Quality of Life Clinical Trial Questionnaire (DQLCTQ), which was designed to assess HRQL among patients with type 1 and type 2 diabetes in multinational clinical trials [43]. The 10 TFS items evaluate how much choice patients have in their decisions concerning meals and physical, social, and other daily activities during the past four weeks [43]. Five questions focus on meals, while the other five focus on activities. Each item is answered on a 5-point Likert scale. The TFS score ranges from 0 to 100, with higher scores indicating greater flexibility. The instrument has demonstrated good internal consistency reliability and discriminant validity [43,44].

### Diabetes Treatment Satisfaction Questionnaire (DTSQ)

The DTSQ was designed to measure satisfaction with diabetes treatment regimens among patients with type 1 or type 2 diabetes [22,45]. The instrument is comprised of eight items, each rated on a 7-point Likert scale ranging from 0 to 6. Six of the items contribute to a treatment satisfaction score, and the other two items assess perceived frequency of hyperglycemia and hypoglycemia. On the satisfaction scale, which ranges from 0 to 36, higher scores indicate greater satisfaction. On the hyperglycemia and hypoglycemia items, higher scores indicate greater problems. The current study used the "status form" of the DTSQ (which measures satisfaction at one point in time), as opposed to the "change form" (which measures change in satisfaction) [46]. The instrument has been used to measure outcomes of diabetes management and clinical trials, and it has been shown to be reliable, and valid, and sensitive to change [22,29,45,46].

### EuroQol EQ-5D

The EQ-5D is a brief questionnaire that is commonly used to provide an estimate of overall health status in large-scale surveys, clinical research, and health economic evaluation [47]. The EQ-5D descriptive system consists of five dimensions of health (mobility, self-care, usual activities, pain/discomfort, and anxiety/depression). Each dimension is assessed by one item with three response choices: no problems, some problems, and severe problems. These five ratings are used to derive the weighted EQ-5D index score, a single score representing overall health with higher scores indicating better health status. An index score of 1 corresponds to perfect health, and 0 corresponds to death, although negative scores representing health states worse than death are possible [48,49]. Reliability, validity, and responsiveness of the EQ-5D have been demonstrated in general population samples as well as samples of patients with a wide range of medical conditions [47]. Mean scores for general samples of patients with type 2 diabetes in previous studies range from roughly 0.69 to 0.77 [50-52]. Scores tend to be somewhat lower among patients with complications, patients being treated with insulin, patients with obesity, and older patients.

### Vitality scale of the SF-36 (medical outcomes study short form-36 item health survey)

The SF-36 was created to collect health status information across a variety of diseases and treatment groups [53,54]. The instrument was designed to be appropriate for use in a variety of settings including clinical practice, clinical research, health policy evaluations, and general population surveys. The SF-36 consists of 8 subscales, but only the 4-item vitality subscale was administered in the current trial to assess energy level and fatigue. The four items are rated on 6-point Likert scales: two items that are worded positively ("Did you feel full of pep"; "Did you have a lot of energy") and two items that are worded negatively ("Did you feel worn out"; "Did you feel tired"). Scores range from 0 to 100, with higher scores reflecting less fatigue and greater energy. Reliability and validity of SF-36 scales have been evaluated in multiple studies and have generally been found to be acceptable [54,55]. Mean subscale scores for patients with type 2 diabetes have typically ranged from approximately 40 to 60 in previous studies, and scores have been shown to improve with treatment [56,57]. Vitality scores have also been shown to decline with the onset of diabetes complications [58].

### Statistical analysis

This analysis was conducted with the per protocol sample, which included all patients who had at least 12 weeks of exposure to study medication and no violations of inclusion/exclusion criteria or discontinuation criteria (e.g., 1.5% increase in HbA_1c_, more than 10 consecutive days of study medication are missed, or a female patient becomes pregnant). For patients who completed the endpoint analysis earlier than week 26, a last observation carried forward (LOCF) approach was used (i.e., substituting data gathered at week 12, 18, or 26).

Categorical demographic and clinical variables are presented in terms of frequency and percents, whereas continuous variables are summarized in terms of means and standard deviations. To evaluate within-group change in each health outcomes measure, paired t-tests were conducted to compare baseline and endpoint scores. To examine differences between the two treatment groups, general linear models were performed, controlling for country and baseline score. The dependent variable in each model was the health outcome measure change score (endpoint – baseline). Separate models were conducted for each instrument's total and subscale scores. Finally, because exenatide was associated with a higher incidence of nausea than insulin glargine [2], change in treatment satisfaction (as measured by the DTSQ) was also assessed separately among subgroups of exenatide-treated and insulin glargine-treated patients who experienced nausea at any time during the trial. Results of all analyses were considered statistically significant at a level of p < 0.05. Because these analyses were considered exploratory, no adjustments for multiple comparisons were made.

## Results

A total of 549 patients were enrolled in the trial. The current analyses were conducted with data from the 455 patients that were considered per protocol (228 exenatide and 227 insulin glargine). Demographic and clinical characteristics of the total sample and two treatment groups are presented in Table 1. The total per protocol sample was primarily Caucasian (79.6%), with slightly more men (55.2%) than women. The mean age was 58.5 years, and patients had type 2 diabetes for a mean of 9.5 years. Mean HbA1_c _and BMI at baseline were 8.3% and 31.5 kg/m^2^, respectively. There were no statistically significant differences at baseline between the two treatment groups in these demographic and clinical variables.

**Table 1:** Demographic and clinical characteristics

**Characteristic**	**Exenatide (N = 228)**	**Insulin Glargine (N = 227)**	**Total (N = 455)**	**p value**^**1**^
Age (mean, SD)	59.4 (8.9)	57.7 (9.4)	58.5 (9.2)	0.06
Gender (N, % male)	125 (54.8%)	126 (55.5%)	251 (55.2%)	0.92
Ethnicity (N, %)				
Caucasian	181 (79.4%)	181 (79.7%)	362 (79.6%)	0.69
Hispanic	37 (16.2%)	35 (15.4%)	72 (15.8%)	
Western Asian	5 (2.2%)	2 (0.9%)	7 (1.5%)	
African Descent	1 (0.4%)	3 (1.3%)	4 (0.9%)	
Native American	0 (0.0%)	1 (0.4%)	1 (0.2%)	
Other	4 (1.8%)	5 (2.2%)	9 (2.0%)	
Duration of Diabetes in years (mean, SD)	9.7 (5.6)	9.2 (5.9)	9.5 (5.7)	0.21
HBA_1c _(mean, SD)	8.3% (0.9%)	8.3% (1.0%)	8.3% (1.0%)	0.55
BMI (mean kg/m^2^, SD)	31.6 (4.5)	31.4 (4.5)	31.5 (4.5)	0.53

^1 ^P values are for comparisons between the 2 treatment groups. T-tests were used for continuous variables, and Fisher's exact tests were used for categorical variables.

Paired t-tests revealed that both treatment groups demonstrated statistically significant baseline to endpoint change on several of the health outcomes instruments (Table 2). Both the exenatide-treated group and the insulin glargine-treated group demonstrated statistically significant improvement in the DSC-R total score (p < 0.0001 for exenatide and p = 0.0002 insulin glargine), the DTSQ satisfaction score (p < 0.0001 for both treatment groups), and the SF-36 Vitality subscale (p = 0.005 for exenatide and p < 0.04 for insulin glargine). Both groups also had statistically significant differences between baseline and endpoint scores on several of the DSC-R subscales (psychology: fatigue, psychology: cognitive, ophthalmology, hypoglycemia, hyperglycemia) as well as the hyperglycemia and hypoglycemia items of the DTSQ. In addition, the insulin glargine group demonstrated significant baseline to endpoint change on the EQ-5D index score and the DSC-R cardiology score.

**Table 2:** Paired t-tests comparing baseline and endpoint scores within each treatment group

**Health Outcomes Measure (mean, SD)**	**Exenatide**			**Insulin Glargine**		
	**Baseline**	**Endpoint**	**p value**	**Baseline**	**Endpoint**	**p value**
DSC-R Overall Score	1.07 (0.83)	0.90 (0.80)	< 0.0001	0.99 (0.78)	0.84 (0.73)	0.0002
EQ-5D Index Score	0.82 (0.22)	0.85 (0.19)	0.08	0.84 (0.22)	0.87 (0.20)	0.049
Diabetes Treatment Flexibility Score	60.37 (22.24)	60.48 (22.33)	0.93	58.85 (22.81)	58.95 (23.37)	0.93
Diabetes Treatment Satisfaction Score	26.41 (7.00)	29.48 (6.12)	< 0.0001	26.31 (6.33)	30.04 (5.21)	< 0.0001
SF-36 Vitality Subscale Score	53.18 (20.87)	56.30 (20.58)	0.005	55.18 (21.35)	57.62 (20.37)	0.04
DSC-R Psychology: Fatigue Score	1.83 (1.26)	1.49 (1.21)	< 0.0001	1.60 (1.29)	1.34 (1.17)	0.0003
DSC-R Psychology: Cognitive Score	1.18 (1.12)	0.99 (1.08)	0.0006	1.14 (1.09)	0.91 (0.99)	0.0001
DSC-R Neurology: Pain Score	0.76 (0.98)	0.70 (0.99)	0.21	0.67 (0.90)	0.63 (0.92)	0.49
DSC-R Neurology: Sensory Score	0.91 (1.07)	0.83 (1.01)	0.10	0.77 (0.94)	0.78 (0.93)	0.83
DSC-R Cardiology Score	0.78 (0.89)	0.71 (0.86)	0.16	0.73 (0.86)	0.61 (0.80)	0.02
DSC-R Ophthalmology Score	0.79 (1.00)	0.62 (0.86)	0.003	0.79 (0.98)	0.64 (0.92)	0.006
DSC-R Hypoglycemia Score	1.09 (1.16)	0.94 (1.09)	0.03	1.10 (1.09)	0.93 (1.00)	0.009
DSC-R Hyperglycemia Score	1.47 (1.31)	1.07 (1.15)	< 0.0001	1.42 (1.25)	1.02 (1.13)	< 0.0001
DTSQ Frequency High Blood Sugar	3.61 (1.76)	2.19 (1.61)	< 0.0001	3.57 (1.67)	2.11 (1.45)	< 0.0001
DTSQ Frequency Low Blood Sugar	1.02 (1.37)	1.36 (1.56)	0.007	0.80 (1.21)	1.50 (1.43)	< 0.0001

Results of general linear models comparing change in health outcomes between the two treatment groups, controlling for country and baseline score, are presented in Table 3. Results of these models indicate that there were no statistically significant differences between groups in the health outcomes instruments.

**Table 3:** Change in health outcomes associated with exenatide and insulin glargine

Health Outcomes Measure	Exenatide			Insulin Glargine			
	N	LS Mean	SE	N	LS Mean	SE	p value^1^
DSC-R Overall Score	223	-0.16	0.04	219	-0.16	0.05	0.96
EQ-5D Index Score	217	0.02	0.01	215	0.03	0.01	0.35
Diabetes Treatment Flexibility Score	222	0.32	1.28	219	-0.46	1.27	0.59
Diabetes Treatment Satisfaction Score	213	3.42	0.43	213	3.85	0.43	0.38
SF-36 Vitality Subscale Score	223	2.41	1.24	220	2.81	1.25	0.78
DSC-R Psychology: Fatigue Score	222	-0.28	0.08	220	-0.31	0.08	0.73
DSC-R Psychology: Cognitive Score	223	-0.24	0.06	220	-0.29	0.06	0.52
DSC-R Neurology: Pain Score	222	-0.04	0.06	219	-0.03	0.06	0.93
DSC-R Neurology: Sensory Score	223	-0.03	0.06	219	0.02	0.06	0.45
DSC-R Cardiology Score	223	-0.08	0.06	219	-0.14	0.06	0.30
DSC-R Ophthalmology Score	222	-0.19	0.06	219	-0.16	0.06	0.68
DSC-R Hypoglycemia Score	221	-0.20	0.07	219	-0.22	0.07	0.81
DSC-R Hyperglycemia Score	223	-0.35	0.07	220	-0.39	0.07	0.58
DTSQ Frequency High Blood Sugar	219	-1.40	0.12	218	-1.48	0.12	0.58
DTSQ Frequency Low Blood Sugar	218	0.37	0.12	216	0.58	0.12	0.13

^1^Comparisons between treatment groups were performed with general linear models. One model was conducted with each health outcomes measure as the dependent variable, controlling for country and baseline score.

Finally, because exenatide has been found to be associated with a higher incidence of nausea than insulin glargine, treatment satisfaction was examined separately among subgroups of patients who experienced nausea at any time during the trial. In the exenatide group, 126 patients reported experiencing nausea at any time during the trial, compared with 22 insulin glargine-treated patients. The subgroup of 126 exenatide-treated patients had mean DTSQ satisfaction scores of 26.9 (SD = 6.8) at baseline and 29.0 (SD = 6.2) at endpoint. A paired t-test found that this improvement (change score = 2.1; SD = 7.4) was statistically significant (t = 3.1, p = 0.002). Findings for the 22 insulin glargine-treated patients were similar. The baseline mean DTSQ satisfaction score was 24.1 (SD = 6.3), and the endpoint score was 30.4 (SD = 4.8). This improvement was also statistically significant (change score = 6.2; SD = 6.3; t = 4.6, p = 0.0001).

## Discussion

The current findings add to previous literature suggesting that, among patients whose glucose levels and symptoms are not adequately controlled by oral medications, the improved efficacy offered by the addition of injectable medication may lead to improved treatment satisfaction and quality of life [57,59,60]. This analysis found that both exenatide and insulin glargine were associated with significant improvements in patient-reported outcomes when added to oral medications among patients with type 2 diabetes. Patients in both treatment groups demonstrated statistically significant baseline-to-endpoint improvement in overall treatment satisfaction as measured by the DTSQ and vitality as measured by a subscale of the SF-36. Both groups also had significant reductions in overall symptom impact and problems with several specific symptom domains as measured by the DSC-R (e.g., fatigue, cognition, ophthalmology, hypoglycemia, hyperglycemia). Insulin glargine-treated patients also had statistically significant improvement in overall HRQL as assessed by the EQ-5D. Some studies have reported that patients with type 2 diabetes on oral medications have greater HRQL than patients on insulin [24,51,61-63]. However, current findings are consistent with other studies showing increased HRQL and patient satisfaction after initiating insulin therapy [57,59,60]. Findings were consistent for both drugs despite different side effect profiles and the fact that exenatide was administered twice daily while insulin glargine was administered once daily.

Analyses comparing patient-reported outcomes of the two drugs found no significant differences between treatment groups despite drug differences in several areas such as weight change, side effect profile, and dose frequency. Although exenatide is associated with increased injections and gastrointestinal side effects compared with insulin glargine, these potential problems did not appear to result in less patient satisfaction among the exenatide-treated patients. It is possible that, for patients who experienced gastrointestinal side effects from exenatide, the weight reduction benefits associated with the drug outweighed its disadvantages, thus resulting in the observed gains in treatment satisfaction. In addition, although increased dosing frequency often leads to reduced patient satisfaction, this finding is not consistent across all diseases and medications [32]. For example, one previous study conducted with patients who had type 2 diabetes found no treatment satisfaction differences between patients receiving once-daily injections and those receiving twice-daily injections [28]. Both current results and these previous findings suggest that dosing frequency may not be of primary importance to patients receiving injectable medication for type 2 diabetes.

Several aspects of the current study design may have limited the ability to detect true differences in patient experience with these two medications. First, is possible that a naturalistic study conducted with less structure than a clinical trial might yield different findings. For example, if patients have less contact with medical professionals, they might be less adherent to a more complicated dosing regimen. A second possible limitation is the relatively brief study duration. In longer trials of these medications, patients have experienced greater weight change than in this 26-week trial [64], and greater weight change is likely to have a stronger impact on treatment satisfaction and vitality. Third, a larger sample size would provide greater statistical power for detecting statistically significant differences between treatment groups, if in fact there are true differences. Finally, the only HRQL instrument administered in this trial was the brief EQ-5D, which may not be sufficiently sensitive to between-treatment group HRQL differences in this population. Perhaps a multidimensional generic HRQL measure or a condition-specific HRQL measure would have been able to detect differences.

Another factor limiting the interpretation of data is that minimally important differences (MIDs) have not been identified for the three condition-specific instruments used in this study (i.e., DSC-R, DTSQ, and TFS). MID is defined as the smallest change score that a patient would perceive as beneficial [65,66]. For patient-reported outcome measures, the minimally important difference (MID) is used as a guideline to interpret whether improvement can be considered clinically significant or meaningful to patients. Although both treatment groups demonstrated statistically significant change in most of the condition-specific scales, it is not known whether these changes are clinically meaningful.

Previous research has identified MIDs of the two generic instruments used in the current study. MIDs have been suggested to be roughly 3 to 5 for the SF-36 and 0.07 for the EQ-5D, although these MIDs were not derived within samples of patients with diabetes [67,68]. Neither treatment group in the current study met the MID criterion for the EQ-5D. On the SF-36 vitality subscale, the exenatide-treated group changed by 3.12 points, which does exceed the lower estimate of MID for this scale, while the insulin glargine group improved by 2.44 points. However, interpretation of treatment effects should not be made based solely on these generic measures because generic instruments tend to be less responsive to change than condition-specific instruments [37]. Therefore, future research on MIDs in the three diabetes-specific measures is necessary in order to estimate the clinical significance of patient-reported improvement in the current study.

Treatment satisfaction is important largely because it is thought to provide an indication of treatment adherence [69-71]. In general, patients who are satisfied with their treatment can be expected to adhere to prescribed treatment regimens more than patients who are unsatisfied. Therefore, patient satisfaction is necessary in order to maximize treatment effectiveness. In sum, current results indicate that both exenatide and insulin glargine were associated with increased treatment satisfaction and vitality as well as decreased symptom burden.

## Competing interests

KS is an employee and stock holder of Eli Lilly and Company. LM was a paid consultant. AO is an employee and stock holder of Eli Lilly and Company. KM was a paid consultant. SK was a paid consultant. RH is an employee and stock holder of Eli Lilly and Company. RB is an employee and stock holder of Eli Lilly and Company.

## Authors' contributions

KS formulated the study hypotheses, guided the project, provided input into data analyses/interpretation, and critically reviewed the manuscript. LM wrote the manuscript and provided input into the statistical analyses and data interpretation. AO provided input into data interpretation and critically reviewed the manuscript. KM performed statistical analyses and critically reviewed the manuscript. SK performed statistical analyses and critically reviewed the manuscript. RH initiated the quality of life study design, provided input into the statistical analyses, and critically reviewed the manuscript. RB provided input into the study design, provided diabetes-related clinical expertise to help interpret the results, and critically reviewed the manuscript. All authors have read and approved the final manuscript.

## References

[B1] Leidy NK, Revicki DA, Geneste B (1999). Recommendations for evaluating the validity of quality of life claims for labeling and promotion. Value in Health.

[B2] Heine RJ, Van Gaal LF, Johns D, Mihm MJ, Widel MH, Brodows RG (2005). Exenatide versus insulin glargine in patients with suboptimally controlled type 2 diabetes: a randomized trial. Ann Intern Med.

[B3] Davis T, Edelman SV (2004). Insulin therapy in type 2 diabetes. Med Clin North Am.

[B4] Oiknine R, Bernbaum M, Mooradian AD (2005). A critical appraisal of the role of insulin analogues in the management of diabetes mellitus. Drugs.

[B5] Gerich JE (2004). Insulin glargine: long-acting basal insulin analog for improved metabolic control. Curr Med Res Opin.

[B6] McKeage K, Goa KL (2002). Spotlight on insulin glargine in type 1 and 2 diabetes mellitus. Treat Endocrinol.

[B7] Buse JB, Henry RR, Han J, Kim DD, Fineman MS, Baron AD (2004). Effects of exenatide (exendin-4) on glycemic control over 30 weeks in sulfonylurea-treated patients with type 2 diabetes. Diabetes Care.

[B8] DeFronzo RA, Ratner RE, Han J, Kim DD, Fineman MS, Baron AD (2005). Effects of exenatide (exendin-4) on glycemic control and weight over 30 weeks in metformin-treated patients with type 2 diabetes. Diabetes Care.

[B9] Giannoukakis N (2003). Exenatide. Amylin/Eli Lilly. Curr Opin Investig Drugs.

[B10] Keating GM (2005). Exenatide. Drugs.

[B11] Kendall DM, Kim D, Poon T, Han J, Schnabel C, Fineman M, Trautmann M, Maggs D (2005). Improvements in cardiovascular risk factors accompanied sustained effects on glycemia and weight reduction in patients with type 2 diabetes treated with exenatide for 82 weeks. Podium presentation at the 65th Scientific Sessions of the American Diabetes Association.

[B12] Kendall DM, Riddle MC, Rosenstock J, Zhuang D, Kim DD, Fineman MS, Baron AD (2005). Effects of exenatide (exendin-4) on glycemic control over 30 weeks in patients with type 2 diabetes treated with metformin and a sulfonylurea. Diabetes Care.

[B13] Nauck MA, Meier JJ (2005). Glucagon-like peptide 1 and its derivatives in the treatment of diabetes. Regul Pept.

[B14] Poon T, Nelson P, Shen L, Mihm M, Taylor K, Fineman M, Kim D (2005). Exenatide improves glycemic control and reduces body weight in subjects with type 2 diabetes: a dose-ranging study. Diabetes Technol Ther.

[B15] de Sonnaville JJ, Snoek FJ, Colly LP, Deville W, Wijkel D, Heine RJ (1998). Well-being and symptoms in relation to insulin therapy in type 2 diabetes. Diabetes Care.

[B16] Heller S (2004). Weight gain during insulin therapy in patients with type 2 diabetes mellitus. Diabetes Res Clin Pract.

[B17] Purnell JQ, Weyer C (2003). Weight effect of current and experimental drugs for diabetes mellitus: from promotion to alleviation of obesity. Treat Endocrinol.

[B18] Albu J, Raja-Khan N (2003). The management of the obese diabetic patient. Prim Care.

[B19] The Diabetes Control and Complications Trial Research Group (DCCT) (1993). The effect of intensive treatment of diabetes on the development and progression of long-term complications in insulin-dependent diabetes mellitus. N Engl J Med.

[B20] Scheen AJ (2000). Treatment of diabetes in patients with severe obesity. Biomed Pharmacother.

[B21] (1998). Intensive blood-glucose control with sulphonylureas or insulin compared with conventional treatment and risk of complications in patients with type 2 diabetes (UKPDS 33). UK Prospective Diabetes Study (UKPDS) Group. Lancet.

[B22] Bradley C, Lewis KS (1990). Measures of psychological well-being and treatment satisfaction developed from the responses of people with tablet-treated diabetes. Diabet Med.

[B23] Lee AJ, Morgan CL, Morrissey M, Wittrup-Jensen KU, Kennedy-Martin T, Currie CJ (2005). Evaluation of the association between the EQ-5D (health-related utility) and body mass index (obesity) in hospital-treated people with Type 1 diabetes, Type 2 diabetes and with no diagnosed diabetes. Diabet Med.

[B24] Redekop WK, Koopmanschap MA, Stolk RP, Rutten GE, Wolffenbuttel BH, Niessen LW (2002). Health-related quality of life and treatment satisfaction in Dutch patients with type 2 diabetes. Diabetes Care.

[B25] Patrick DL, Chiang YP (2000). Measurement of health outcomes in treatment effectiveness evaluations: Conceptual and methodological challenges. Med Care.

[B26] Edwards CM, Stanley SA, Davis R, Brynes AE, Frost GS, Seal LJ, Ghatei MA, Bloom SR (2001). Exendin-4 reduces fasting and postprandial glucose and decreases energy intake in healthy volunteers. Am J Physiol Endocrinol Metab.

[B27] Maggs D, Kim D, Holcombe J, Han J, Shen L, Ruggles J, Fineman M (2005). Exenatide-induced reductions in A1C and body weight in long-term trials are not explained by gastrointestinal side effects. 65th Annual Scientific Session of the American Diabetes Association.

[B28] Brod M, Perwien A, Adler L, Spencer T, Johnston J (2005). Conceptualization and assessment of Quality of Life for adults with Attention-Deficit/Hyperactivity Disorder. Primary Psychiatry.

[B29] (2002). Training in flexible, intensive insulin management to enable dietary freedom in people with type 1 diabetes: dose adjustment for normal eating (DAFNE) randomised controlled trial. BMJ.

[B30] Masoli M, Weatherall M, Holt S, Beasley R (2004). Budesonide once versus twice-daily administration: meta-analysis. Respirology.

[B31] Pellock JM, Smith MC, Cloyd JC, Uthman B, Wilder BJ (2004). Extended-release formulations: simplifying strategies in the management of antiepileptic drug therapy. Epilepsy Behav.

[B32] Richter A, Anton SE, Koch P, Dennett SL (2003). The impact of reducing dose frequency on health outcomes. Clin Ther.

[B33] Brommels M, Sintonen H (2001). Be generic and specific: quality of life measurement in clinical studies. Ann Med.

[B34] Engstrom CP, Persson LO, Larsson S, Sullivan M (2001). Health-related quality of life in COPD: why both disease-specific and generic measures should be used. Eur Respir J.

[B35] Leong KP, Yeak SC, Saurajen AS, Mok PK, Earnest A, Siow JK, Chee NW, Yeo SB, Khoo ML, Lee JC, Seshadri R, Chan SP, Tang CY, Chng HH (2005). Why generic and disease-specific quality-of-life instruments should be used together for the evaluation of patients with persistent allergic rhinitis. Clin Exp Allergy.

[B36] Vickrey BG, Hays RD, Genovese BJ, Myers LW, Ellison GW (1997). Comparison of a generic to disease-targeted health-related quality-of-life measures for multiple sclerosis. J Clin Epidemiol.

[B37] Wiebe S, Guyatt G, Weaver B, Matijevic S, Sidwell C (2003). Comparative responsiveness of generic and specific quality-of-life instruments. J Clin Epidemiol.

[B38] Coons SJ, Rao S, Keininger DL, Hays RD (2000). A comparative review of generic quality-of-life instruments. Pharmacoeconomics.

[B39] Graue M, Wentzel-Larsen T, Hanestad BR, Batsvik B, Sovik O (2003). Measuring self-reported, health-related, quality of life in adolescents with type 1 diabetes using both generic and disease-specific instruments. Acta Paediatr.

[B40] Guyatt GH, Feeny DH, Patrick DL (1993). Measuring health-related quality of life. Ann Intern Med.

[B41] Grootenhuis PA, Snoek FJ, Heine RJ, Bouter LM (1994). Development of a type 2 diabetes symptom checklist: a measure of symptom severity. Diabet Med.

[B42] Gulliford MC, Mahabir D (1999). Relationship of health-related quality of life to symptom severity in diabetes mellitus: a study in Trinidad and Tobago. J Clin Epidemiol.

[B43] Shen W, Kotsanos JG, Huster WJ, Mathias SD, Andrejasich CM, Patrick DL (1999). Development and validation of the Diabetes Quality of Life Clinical Trial Questionnaire. Med Care.

[B44] Hayes CP, Bowman L (2003). Reliability and validity of the Treatment Flexibility Scale. Qual Life Res.

[B45] Bradley C, Bradley C (1994). The Diabetes Treatment Satisfaction Questionnaire: DTSQ. Handbook of Psychology and Diabetes: A guide to psychological measurement in diabetes research and practice.

[B46] Bradley C, Speight J (2002). Patient perceptions of diabetes and diabetes therapy: assessing quality of life. Diabetes Metab Res Rev.

[B47] Brooks R, Rabin R, de Charro F (2003). The Measurement and Valuation of Health Status using EQ-5D: A European Perspective.

[B48] Krabbe P, Weijnen T, Brooks R, Rabin R and de Charro F (2003). Guidelines for analysing and reporting EQ-5D outcomes. The Measurement and Valuation of Health Status using EQ-5D: A European Perspective.

[B49] McDowell I, Newell C (1996). Measuring Health: A guide to rating scales and questionnaires.

[B50] Clarke P, Gray A, Holman R (2002). Estimating utility values for health states of type 2 diabetic patients using the EQ-5D (UKPDS 62). Med Decis Making.

[B51] Koopmanschap M (2002). Coping with Type II diabetes: the patient's perspective. Diabetologia.

[B52] Bech P, Moses R, Gomis R (2003). The effect of prandial glucose regulation with repaglinide on treatment satisfaction, wellbeing and health status in patients with pharmacotherapy naive Type 2 diabetes: a placebo-controlled, multicentre study. Qual Life Res.

[B53] Ware JEJ, Sherbourne CD (1992). The MOS 36-item short-form health survey (SF-36). I. Conceptual framework and item selection. Med Care.

[B54] Ware JEJ, Snow KK, Kosinski M, Gandek B (1993). Applications of the SF-36. SF-36 Health Survey: Manual and Interpretation Guide.

[B55] McHorney CA, Ware JEJ, Lu JF, Sherbourne CD (1994). The MOS 36-item Short-Form Health Survey (SF-36): III. Tests of data quality, scaling assumptions, and reliability across diverse patient groups. Med Care.

[B56] Ibrahim IA, Beich J, Sidorov J, Gabbay R, Yu L (2002). Measuring outcomes of type 2 diabetes disease management program in an HMO setting. South Med J.

[B57] Reza M, Taylor CD, Towse K, Ward JD, Hendra TJ (2002). Insulin improves well-being for selected elderly type 2 diabetic subjects. Diabetes Res Clin Pract.

[B58] Ahroni JH, Boyko EJ (2000). Responsiveness of the SF-36 among veterans with diabetes mellitus. J Diabetes Complications.

[B59] Fischer JS, McLaughlin T, Loza L, Beauchamp R, Schwartz S, Kipnes M (2004). The impact of insulin glargine on clinical and humanistic outcomes in patients uncontrolled on other insulin and oral agents: an office-based naturalistic study. Curr Med Res Opin.

[B60] Wilson M, Moore MP, Lunt H (2004). Treatment satisfaction after commencement of insulin in Type 2 diabetes. Diabetes Res Clin Pract.

[B61] Jacobson AM (1997). Quality of Life in Patients With Diabetes Mellitus. Semin Clin Neuropsychiatry.

[B62] Maddigan SL, Majumdar SR, Toth EL, Feeny DH, Johnson JA (2003). Health-related quality of life deficits associated with varying degrees of disease severity in type 2 diabetes. Health Qual Life Outcomes.

[B63] Pibernik-Okanovic M, Szabo S, Metelko Z (1998). Quality of life following a change in therapy for diabetes mellitus. Pharmacoeconomics.

[B64] Blonde L, Klein EJ, Han J, Zhang B, Mac SM, Poon TH, Taylor KL, Trautmann ME, Kim DD, Kendall DM (2006). Interim analysis of the effects of exenatide treatment on A1C, weight and cardiovascular risk factors over 82 weeks in 314 overweight patients with type 2 diabetes. Diabetes Obes Metab.

[B65] Guyatt GH, Osoba D, Wu AW, Wyrwich KW, Norman GR (2002). Methods to explain the clinical significance of health status measures. Mayo Clin Proc.

[B66] Jaeschke R, Singer J, Guyatt GH (1989). Measurement of health status. Ascertaining the minimal clinically important difference. Control Clin Trials.

[B67] Hays RD, Morales LS (2001). The RAND-36 measure of health-related quality of life. Ann Med.

[B68] Walters SJ, Brazier JE (2005). Comparison of the minimally important difference for two health state utility measures: EQ-5D and SF-6D. Qual Life Res.

[B69] Revicki DA (2004). Patient assessment of treatment satisfaction: methods and practical issues. Gut.

[B70] Shikiar R, Flood E, Siddique R, Howell J, Dodd SL (2005). Development and validation of the Gastroesophageal Reflux Disease Treatment Satisfaction Questionnaire. Dig Dis Sci.

[B71] Speight J (2005). Assessing patient satisfaction: concepts, applications, and measurement. Value in Health.

